# Supplementary material for: OsOSCA1.1 Mediates Hyperosmolality and Salt Stress Sensing in Oryza sativa
Source: Biology (Basel). 2022 Apr 28;11(5):678. doi: 10.3390/biology11050678 (PMC9138581; doi:10.3390/biology11050678)

Supplementary Information

Supplementary Table S1. Primers used in this study.

Supplementary Table S2. Summary of RNA sequencing data from 30-day-old ZH11 and *ososca1.1* plants in response to hyperosmolality stress (250 mM sorbitol) and salt stress (125 mM NaCl) for 24 h.

Supplementary Table S3. Differentially expressed genes (DEGs) enriched in hormone responses, transcription regulation, and phosphorylation Gene Ontology (GO) analysis terms.

Supplementary Table S4. Numbers of fragments per kilobase of exon per million mapped fragments (FPKM) for OsOSCA1.1-regulated genes.

Supplementary Table S5. Distributions of *cis*-regulatory elements in the 2,000-bp promoter region of OsOSCA1.1-regulated genes.

Supplementary Figure S1. Characterisation of the rice T-DNA insertion mutant *ososca1.1,* and *OsOSCA1.1*-overexpressing *ososca1.1* lines 1 and 2. (**A**) Schematic diagram of T-DNA insertion in the *Oryza sativa* genome of *OsOSCA1.1* (RMD_ITL-03Z11CJ17). Open box, UTR; black box, exon; line, intron. Bar = 500 bp. (**B**) Genotypes of *ososca1.1* were identified by polymerase chain reaction (PCR). Wild-type rice (ZH11) was used as the control. *OsActin1* (Os03g0836000) was used as the experimental control. LP, LB, and RP primers used for PCR are listed in Table S1. (**C**) Semi-quantitative PCR analysis to detect *OsOSCA1.1* levels in *OsOSCA1.1*-overexpressing *ososca1.1* lines-1 and -2. Wild-type rice (ZH11) and *ososca1.1* was used as the control. *OsActin1* was used as the experimental control.

Supplementary Figure S2. Stomatal density in leaves of ZH11, *ososca1.1*, and

OsOSCA1.1-overexpressing *ososca1.1* lines 1 and 2. The leaves were photographed with Environmental scanning electron micrographs, and the numbers of stomata per mm^2 was counted (n = 30 leaves). NS indicates no significant differences at $P < 0.05$ (one-way ANOVA).

Supplementary Figure S3. Volcano plots of the total gene expression profiles of ZH11 and *ososca1.1* following treatment with 250 mM sorbitol and 125 mM NaCl for 24 h. Dots represent mean expression levels of individual genes obtained from a normalised RNA sequencing dataset. A fold change = |2.0| and adjusted $P < 0.05$ were the cut-offs for classification as differentially expressed genes (DEGs; genes denoted by grey lines) Downregulated and upregulated genes are denoted by blue and yellow dots, respectively).

Supplementary Figure S4. The enrichment analysis in “Cellular Component” category of GO with OsOSCA1.1-regulated genes in 30-day-old rice shoots in response to hyperosmolality and salt stress treatment. (**A**) Six terms enriched with sorbitol response genes. (**B**) Nine terms enriched with osmotic stress response genes. (**C**) The top 10 terms enriched with NaCl response genes were displayed. GO term information for DEGs was extracted from BLAST results against the SWISS-PROT database.

Supplementary Figure S5. The enrichment analysis in “Molecular Function” category of GO with OsOSCA1.1-regulated genes in 30-day-old rice shoots in response to hyperosmolality and salt stress treatment. (**A**) Sorbitol response genes. (**B**) Osmotic stress response genes. (**C**) NaCl response genes. GO term information for DEGs was extracted from BLAST results against the SWISS-PROT database and the top 10 terms are displayed.

Supplementary Table S1. The primer used in this study.

Primer name	Sequence (5'-3')
For genotype	
LB	AATCCAGATCCCCCGAATTA
LP	TAGGGTATCCTTCCGCATTG
RP	GCTCCTTGTTCTCCCTGTTG
Actin-F	AGTGGTCGTACAACAGGTA
Actin-R	TCTTCATTAGGCAGTCAGT
For semi-quantitate PCR	
OsOSCA1.1-F	CTATGTGCTGTTACGGGAGTAT
OsOSCA1.1-R	CAACCTGGTGCGTAAGATAATG
For cloning *OsOSCA1.1*	
OsOSCA1.1-5'	ATGGCTACTATTCAAGATATAGGT
OsOSCA1.1-3'	TAGCCGTTGATCATTTACAATTTC

Supplementary Table S2. Summary of transcriptome sequence data of 30-day-old ZH11 and *ososca1.1* plants in response to hyperosmolality stress (250 mM sorbitol) and salt stress (125 mM NaCl) for 24 h treatment.

Sample name	Total clean reads	Mapping rate	Total gene number	Description
**CZH11L1**	44,068,846	0.9736	28755	ZH11-1
**CZH11L2**	44,943,488	0.9733	28531	ZH11-2
**COSR1L1**	46,702,630	0.9724	29053	*ososca1.1-1*
**COSR1L2**	45,464,294	0.9724	29076	*ososca1.1-2*
**SZH11L1**	46,929,076	0.9752	28827	ZH11-1, sorbitol treatment
**SZH11L2**	46,439,360	0.9758	28862	ZH11-2, sorbitol treatment
**SOSR1L1**	44,536,098	0.9732	28423	*ososca1.1-1*, sorbitol treatment
**SOSR1L2**	46,560,480	0.9697	28179	*ososca1.1-2*, sorbitol treatment
**NZH11L1**	40,665,524	0.9758	28936	ZH11-1, NaCl treatment
**NZH11L2**	45,670,684	0.9756	29077	ZH11-2, NaCl treatment
**NOSR1L1**	41,216,960	0.9748	27929	*ososca1.1-1*, NaCl treatment
**NOSR1L2**	41,997,214	0.9737	28044	*ososca1.1-2*, NaCl treatment

Supplementary Table S3. Listed DEGs enriched in “hormone response”, “transcription regulation” and “phosphorylation” terms with GO analysis (excel format).

Supplementary Table S4. The FPKM value of three groups of OsOSCA1.1-regulated genes (excel format).

Supplementary Table S5. The distributions of cis-regulatory elements in the 2000-bp promoter region of three groups of OsOSCA1.1-regulated genes.

Groups	DEG numbers	ABRE (%)	ARE (%)	AuxRR-core (%)	DRE (%)	LTR (%)	MYB (%)	MYC (%)	P-box (%)	TATC-box (%)	TGA-element (%)
Sorbitol response genes	Up-regulated (119)	113 (94.96)	106 (89.08)	17 (14.29)	59 (49.58)	65 (54.62)	117 (98.32)	115 (96.64)	37 (31.09)	15 (12.61)	51 (42.86)
	Down-regulated (108)	93 (86.11)	85 (78.70)	21 (19.44)	63 (58.33)	57 (52.78)	106 (98.15)	104 (96.30)	38 (35.19)	17 (15.74)	34 (31.48)
Osmotic stress response genes	Up-regulated (70)	60 (85.71)	57 (81.43)	12 (17.14)	34 (48.57)	29 (41.43)	70 (100)	69 (98.57)	17 (24.29)	16 (22.86)	24 (34.29)
	Down-regulated (56)	54 (96.43)	46 (82.14)	9 (16.07)	33 (58.93)	26 (46.63)	56 (100)	55 (98.21)	15 (26.79)	11 (19.64)	19 (33.93)
NaCl response genes	Up-regulated (79)	74 (93.67)	64 (81.01)	9 (11.39)	40 (50.63)	33 (41.77)	79 (100)	77 (97.47)	29 (36.71)	11 (13.92)	30 (37.97)
	Down-regulated (82)	72 (87.80)	70 (85.37)	16 (19.51)	50 (60.98)	36 (43.90)	80 (97.56)	80 (97.56)	33 (40.24)	17 (20.73)	29 (35.37)

Supplementary Figure S1

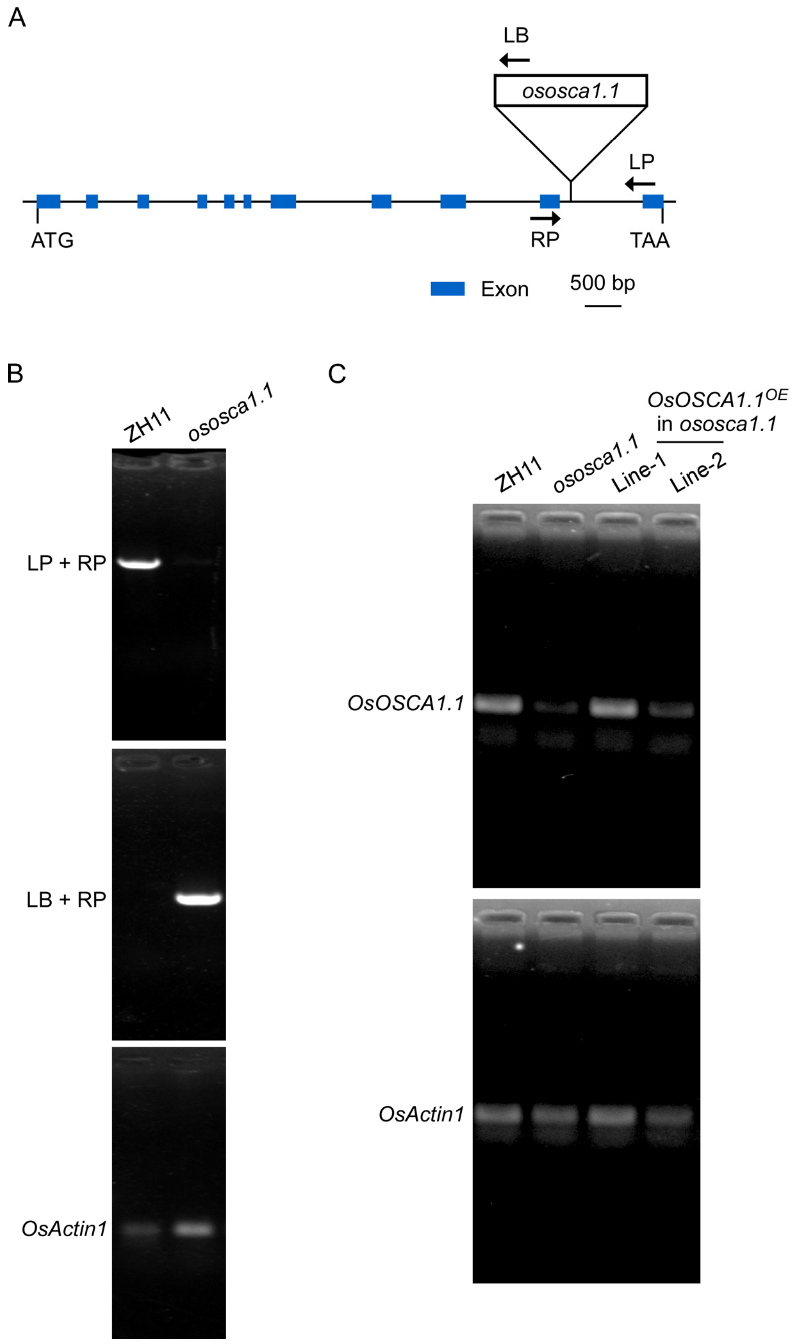

Supplementary Figure S2

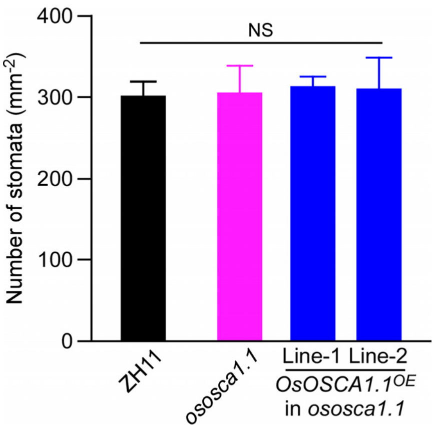

Supplementary Figure S3

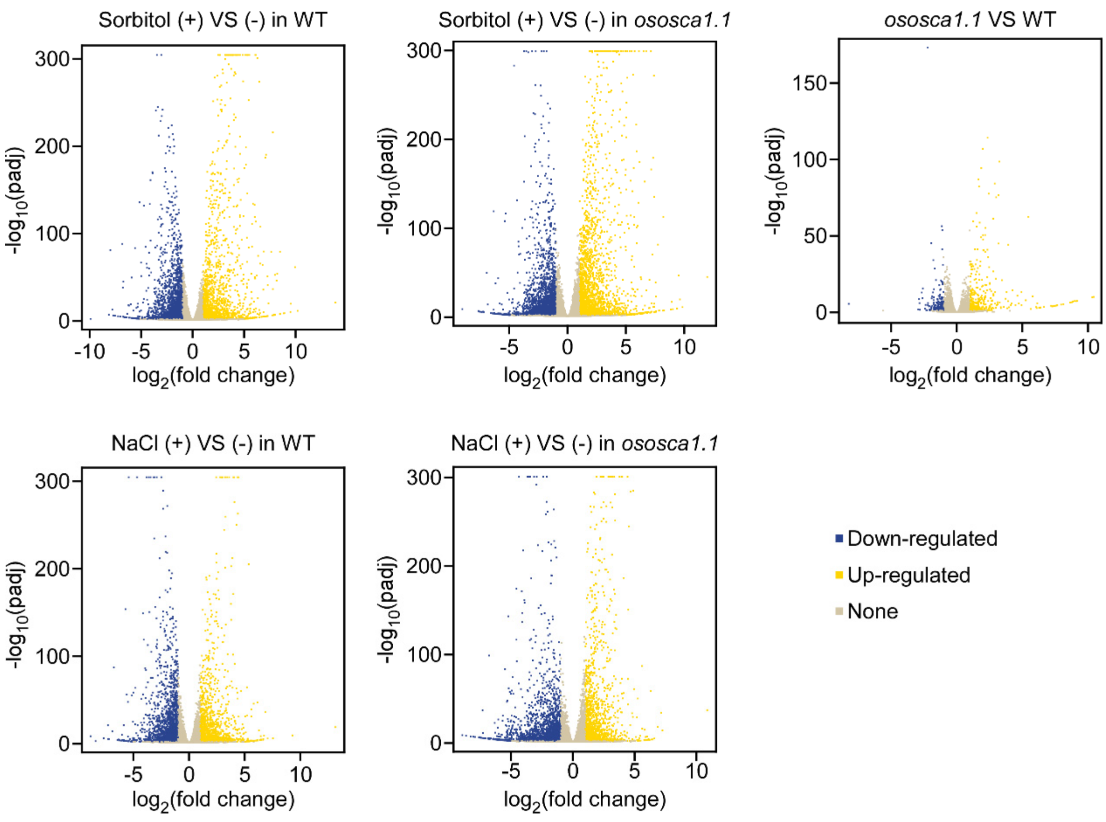

Supplementary Figure S4

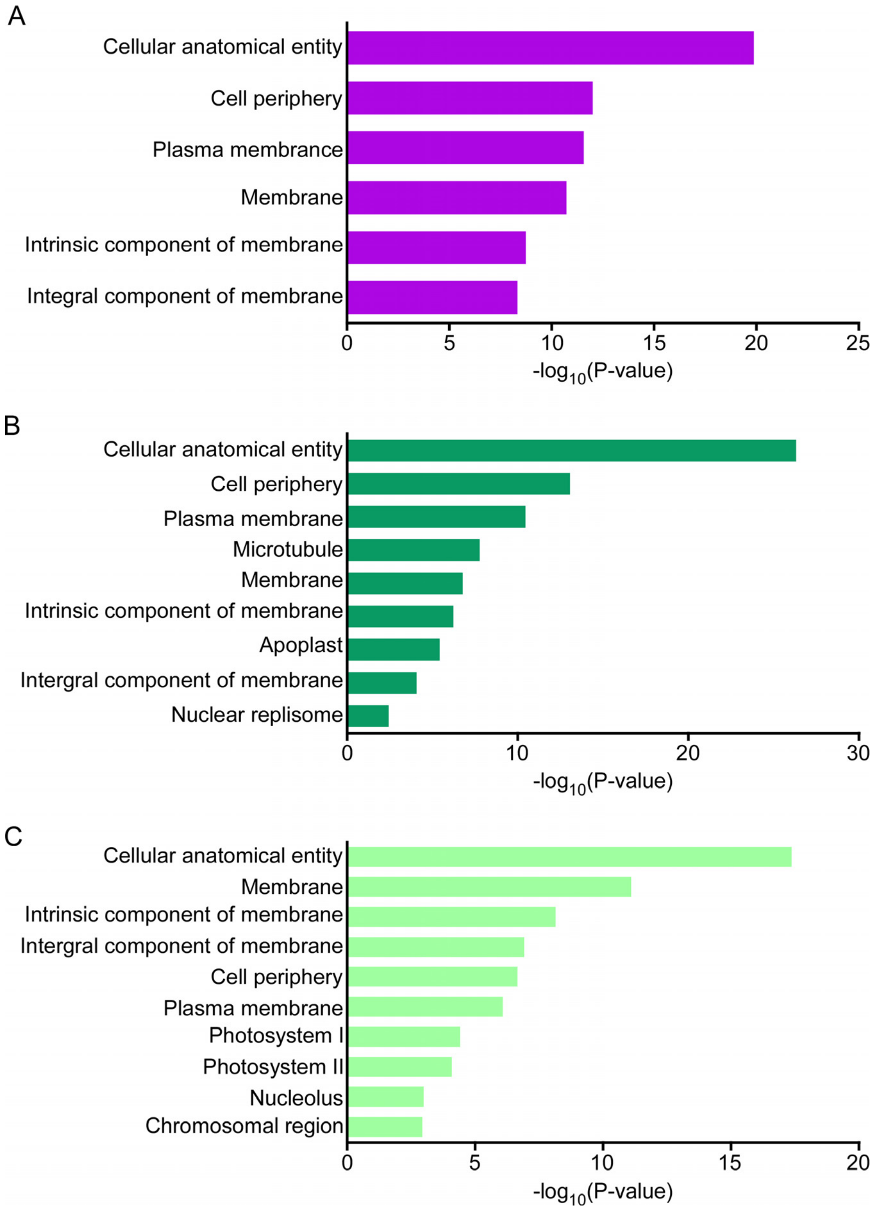

Supplementary Figure S5

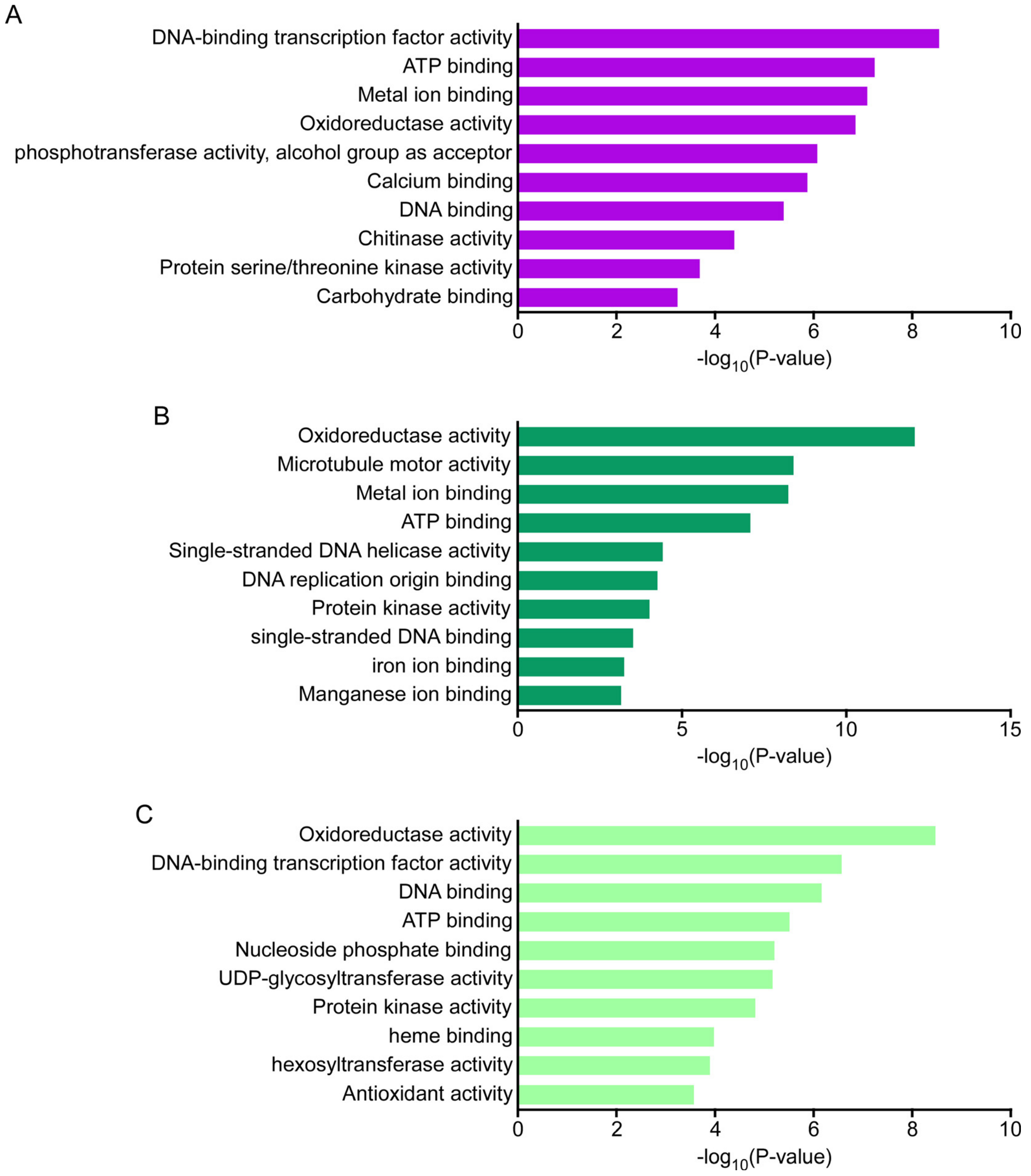

Supplement: Supplementary file 1 [file biology-11-00678-s001.zip › Supplementary Information.pdf]
